# Exosome Loaded in Microneedle Patch Ameliorates Renal Ischemia–Reperfusion Injury in a Mouse Model

**DOI:** 10.1155/sci/3106634

**Published:** 2025-01-15

**Authors:** Samin Taghavi, Somayeh Keshtkar, Mozhgan Abedanzadeh, Mehrdad Hashemi, Reza Heidari, Samira Sadat Abolmaali, Mahintaj Dara, Mahdokht Hossein Aghdaei, Alireza Sabegh, Negar Azarpira

**Affiliations:** ^1^Transplant Research Center, Shiraz University of Medical Sciences, Shiraz, Iran; ^2^Department of Genetics, Faculty of Advanced Science and Technology, Tehran Medical Sciences, Islamic Azad University, Tehran, Iran; ^3^Molecular Dermatology Research Center, Shiraz University of Medical Sciences, Shiraz, Iran; ^4^Pharmaceutical Nanotechnology Department and Center for Nanotechnology in Drug Delivery, Shiraz University of Medical Sciences, Shiraz, Iran; ^5^Farhikhtegan Medical Convergence Sciences Research Center, Farhikhtegan Hospital Tehran Medical Sciences, Islamic Azad University, Tehran, Iran; ^6^Pharmaceutical Sciences Research Center, Faculty of Pharmacy, Shiraz University of Medical Sciences, Shiraz, Iran; ^7^Stem Cells Technology Research Center, Shiraz University of Medical Sciences, Shiraz, Iran

**Keywords:** exosome, mesenchymal stem cell, microneedle patch, renal ischemic injury

## Abstract

**Introduction:** Renal dysfunction due to ischemia–reperfusion injury (IRI) is a common problem after kidney transplantation. In recent years, studies on animal models have shown that exosomes derived from mesenchymal stem cells (MSC-Exo) play an important role in treating acute kidney injury (AKI) and promoting tissue repair. The microneedle patch provides a noninvasive and targeted delivery system for exosomes. The purpose of this innovative approach is to combine MSC-Exo with microneedle patches.

**Method:** Exosomes were isolated from MSCs, characterized, and placed in the prepared microneedle patch. Then this construct was applied to the IRI mice model. After 7 days, the gene expression of miR-34a and its targets B-cell lymphoma-2 (BCL-2) and BCL-2-associated X (BAX), along with reactive oxygen species (ROS) and lipid peroxidation (LPO) production, was investigated. Additionally, renoprotection was evaluated for measuring blood urea nitrogen (BUN) and creatinine (Cr) and histopathology detection.

**Results:** After using microneedle patches containing exosomes, the reduction of miR-34a and BAX and enhancement of BCL-2 were observed. Moreover, treatment by this construct decreased the production of ROS, LPO, BUN, and Cr and improved tissue damage.

**Conclusion:** The use of a microneedle patch containing exosomes is a noninvasive method that enables the release of exosomes in a slow manner. In comparison to exosome injection alone, microneedle patch-exosome treatment offers a longer and more targeted effect that improves renal IRI dysfunction and reduces tissue damage, potentially facilitating the clinical application of exosomes and improving graft survival.

## 1. Introduction

Renal ischemia and reperfusion are pathologic situations, characterized by the initial limitation of blood supply followed by reperfusion and reoxygenation [1]. It is one of the leading causes of acute kidney injury (AKI) which is associated with high morbidity and death. Ischemia–reperfusion injury (IRI) disturbs the cellular redox balance leading to the production of exorbitant reactive oxygen species (ROS). Renal reperfusion is inclusive of mitochondria damage, reduction of energy, apoptosis, and necrosis [2]. IRI is inevitable in the donation process when the donor's kidney experiences ischemia followed by reperfusion immediately after the transplant [3]. Hence, the occurrence of IRI poses a significant clinical hurdle in both native and transplanted kidneys, presenting a scarcity of efficacious therapeutic options.

Numerous investigations have provided evidence that mesenchymal stem cells (MSCs) exhibit therapeutic characteristics due to their capacity to release a wide range of functional factors that play a role in tissue repair and immune regulation. Previous research has demonstrated that MSCs can alleviate kidney damage and enhance kidney function following IRI. These findings indicate that MSCs can effectively restore kidney tissue through the paracrine effects they exert [4, 5]. MSCs possess the ability to secrete a variety of paracrine factors, including soluble growth factors and extracellular vesicles such as exosomes. Exosomes are 30- and 150-nm vesicles that are released into the extracellular environment following the fusion of intracellular vesicles with the plasma membrane. Exosomes are lipid bilayer vesicles that contain kinds of lipids, proteins, messenger RNA (mRNA), and microRNA (miRNA) that participate in cell-to-cell interaction and can transfer biologically active contents to target cells upon binding to the cell membrane. Researchers have been drawn to the significant function of exosomes in the paracrine secretion of MSCs that can result in immune modulation, amelioration of tissue injury, and a decrease in fibrosis [6–9]. Studies showed that exosomes derived from MSCs (MSC-Exo) significantly attenuated pathological kidney damage and could alleviate tissue injuries and apoptosis after IRI [10–14]. However, direct administration of exosomes into the damaged area does not provide sufficient retention time to achieve adequate recovery results. Furthermore, repeated injections of exosomes may lead to an increased risk of secondary damage and reduced therapeutic benefits. Recent research has suggested that microneedle patches exhibit remarkable efficacy as strategies for long-term in situ delivery. Microneedle patches have emerged as a promising platform for delivering drugs, vaccines, and other therapeutics painless and minimally invasive. The microneedles can be made from a variety of materials, including metals, polymers, and biodegradable materials, and can be designed to penetrate different layers of tissue. Dissolving microneedles is a particularly exciting development, as they can dissolve upon insertion and release the drug's prolonged delivery into the tissue, at the same time eliminating the need for needle removal and disposal [9–11]. Hence, in this research, we intend to investigate the effect of the slow release of MSC-Exo by using a new microneedle patch on the IRI in the kidney.

## 2. Materials and Methods

### 2.1. MSC Isolation and Characterization

Human Wharton's jelly-derived MSCs (WJ-MSCs) were isolated from the umbilical cords obtained from cesarean section delivery mothers after obtaining the consent form in accordance with the Shiraz University of Medical Sciences institutional ethics committee (IR.IAU.PS.REC.1401.274). Briefly, the umbilical cord was divided into smaller pieces, Wharton's jelly was removed and placed in DMEM-F12 culture medium (Gibco, Germany) along with 10% fetal bovine serum (FBS) (Gibco, Germany) and 1% penicillin/streptomycin (Pen/Strep) (Sigma Germany) and kept in an incubator with 5% CO_2_ at 37°C. When the cells reached a density above 80%, the cells were passaged by 0.25% trypsin-EDTA (Gibco, Germany). To characterize WJ-MSCs, the isolated cells were investigated for specific surface markers FITC human leukocyte antigen (HLA-DR) (Cat.N:307632), CD34 (Cat N:343503), CD44 (Cat N:338804), and CD90 (Cat N: 328108) (BioLegend, USA) by FACS Calibur flow cytometer (Becton Dickinson, USA).

### 2.2. Exosome Isolation and Characterization

After the cells reached the fourth passage with a density above 80%, the culture medium was removed and replaced with a serum-free culture medium. After 48 h, the conditioned media were collected, and exosomes were separated by using an EXOCIB isolation kit (CIB Biotech., Iran) with some modifications. In brief, the collected conditioned media were centrifuged at 300 g for 15 min and 2000 g for 30 min to eliminate any cells and debris. Then the remaining supernatant was filtered by a 0.22-µ filter and was placed in a 100-kDa Amicon filter (Merc, Germany) and centrifuged at 3500 g for 15 min to remove the smaller vesicles and obtain a more concentrated medium. Then the last remaining supernatant was mixed with reagent A of the EXOCIB kit and overnight incubated at 4°C, and then this mixed content was centrifuged at 3000 g for 40 min to collect exosomes at the bottom of the bottle. The isolated exosomes were kept at −80°C. The exosome content was determined with a BCA protein assay kit (Thermo Scientific Pierce, USA). A dynamic light scattering (DLS) method was used to measure and evaluate the diameter of the particles (Zetasizer 3000HSA, Malvern Instruments, UK). Exosome morphology was evaluated with a transmission electron microscope (TEM) (Zeiss, LEO 906E, Germany). Moreover, the exosomes were investigated for specific surface markers including PE CD9 (Cat N: 555372), CD63 (Cat N: 556020), and CD81 (Cat N: 555676) (BD Bioscience, Belgium) by flow cytometry.

### 2.3. Preparation of Microneedle Patch Containing Exosome

**Figure 1:**
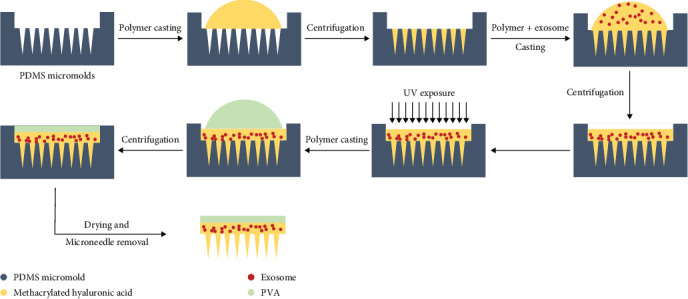
Exosome microneedle patch preparation procedure.

Before the use of microneedle micromolds, empty microneedle molds were exposed to the UVC light, for 20 min, under the laminar air flow cabinet, to eliminate the potential and unwanted microbial contaminations. Then, a 15-μl aqueous solution of methacrylated hyaluronic acid (HA) (3%) containing a photoinitiator was added to the mold and centrifuged for 50 min at 1500 rpm. Then the 100 µg/mL extracted exosmes were mixed with 30 μl of polymer and added to the mold, following the centrifugation for 50 min at 1500 rpm. Then, the polymer casted microneedles were exposed to the 365 nm UV light to prepare the photo-crosslinked hydrogel-based microneedle. After that, 50 μl of 7.5% polyvinyl alcohol (PVA) aqueous solution was added on the top of the photo-crosslinked microneedle and centrifuged for 10 min at 1500 rpm to provide the microneedle's backing cover. Following the dryness of hydrogel-based microneedles, the prepared microneedle was removed gently, and further evaluations were performed. Free microneedles without extracted exosomes were made and used as a microneedle patch group.  Figure 1 demonstrates the microneedle preparation procedure.

### 2.4. IRI Model Development in Mice

Male BALB/c mice (*n* = 20, weighing 23–26 g) were purchased from the Laboratory Animal Breeding Center, Shiraz University of Medical Sciences, Shiraz, Iran. IRI was induced by clamping of the left renal vessel for 40 min along with the right kidney nephrectomy, using the previously used method [15]. The mice were randomly divided into five groups. The first group was the control group (sham) which only underwent nephrectomy. The second group was mice with IRI. The third group was IRI mice in which they received microneedle patches on their kidneys (patch). The fourth and fifth groups were IRI mice that received a direct injection of exosomes (Exo-injection) and microneedle patches containing exosomes into the kidneys (Exo-patch), respectively. The final amount of exosome received in both groups was 100 µg/mL for each mouse. For further molecular and pathological considerations, mice were euthanized after 7 days.

### 2.5. Biochemistry Assay

For evaluation of creatinine (Cr) and blood urea nitrogen (BUN), blood was taken from the hearts and centrifuged at 3000 g, 10 min, 4°C to prepare serum. Then Cr and BUN were measured with commercial kits (Pars Azmun, Tehran, Iran) and a Mindray BS-200 autoanalyzer (Mindray chemistry analyzers for low-volume laboratories, Guangzhou, China).

### 2.6. ROS and Lipid Peroxidation (LPO) Assay

To evaluate the production of ROS, 2,7-dichlorofluorescein diacetate (DCFH-DA) (Sigma, Germany) was used. The fluorescence intensity was detected with a microplate reader in absorption 485 nm (FLUOstar Omega, BMG Labtech, Germany). For the LPO assay, thiobarbituric acid reactive substances (TBARS) were measured in kidney tissue, and the absorbance was read at *λ* = 532 nm (Epoch plate reader, Bio-Tek Instruments, Highland Park, USA). The data of ROS and LPO were normalized with total protein measured by Bradford reagent (Thermo Fisher Scientific, USA) [16, 17].

### 2.7. Kidney Histopathology

The half of kidney tissues were fixed in a formalin solution. Then, 5-μm paraffin-embedded sections were prepared and deparaffinized in dimethylbenzene. Next, the slides were dehydrated in ethanol solutions and finally stained with hematoxylin and eosin (H&E) and observed with a light microscope (Olympus CX21, Japan). Histopathological changes were scored based on 0, 0%; 1, <30%; 2, 31% to 60%; and 3, 61% to 100% [18].

### 2.8. Gene Expression Evaluation by Real-Time PCR

To evaluate the gene expression, total RNA from kidney tissues was extracted by TRIzol reagent (Life Technologies, France). Then, cDNA was synthesized with PrimeScript RT Reagent Kit (Addbio, Korea). The following primers were designed using the NCBI Primer-BLAST tool, mouse B-cell lymphoma-2 (BCL-2) (F: GTGGATGACTGAGTACCTGAAC; R: TCAACCAGACATGCACCTAC), mouse BCL-2-associated X (BAX) (F: CAATATGGAGCTGCAGAGGA; R: GAAGTTGCCATCAGCAAACA), mouse miR-34a (F: TCGCTTGGCAGTGTCTTAGCT; R: UGGCTGUGUCUUTGCUGGUUGU), and mouse beta-actin (F: AGTGTGACGTTGACATCCGT; R: TGCTAGGAGCCAGAGCAGTA), as a housekeeping gene. Finally, real-time RT-PCR was applied for the evaluation of relative gene expression with SYBR Premix Ex TaqTM II Kit (Takara, Japan, #RR820L), using Applied Biosystems StepOnePlus Real-Time PCR. The fold changes were calculated by 2^−*ΔΔ*CT^ for each gene.

### 2.9. Statistical Analysis

The results were shown as mean ± SD. The comparisons between the two groups were evaluated by the unpaired Student's *t*-test. To compare multiple groups, a one-way analysis of variance (ANOVA) was used. The graphs were drawn in GraphPad Prism software (version 9.5.1, San Diego, California). A *p* value below 0.05 was considered statistically significant.

## 3. Results

### 3.1. The MSC Characterization

**Figure 2:**
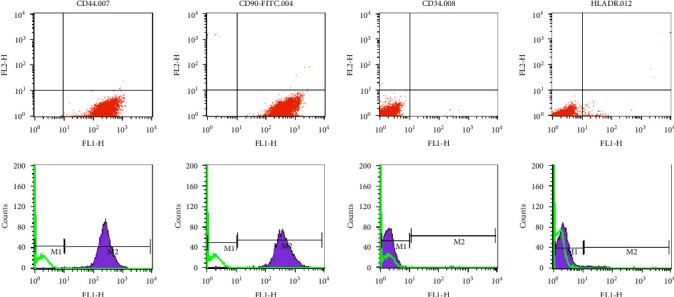
Characterization of WJ-MSCs. Flow cytometry histogram of WJ-MSCs for CD44, CD90, CD34, and HLA-DR surface markers. Green histograms indicate isotype control, whereas purple histograms indicate the signals for each specific marker. HLA-DR, human leukocyte antigen; WJ-MSCs, Wharton's jelly-derived mesenchymal stem cells.

The cells were positive for surface markers CD44 and CD90 and negative for markers CD34 and HLA-DR (Figure 2).

### 3.2. Exosome Characterization

**Figure 3:**
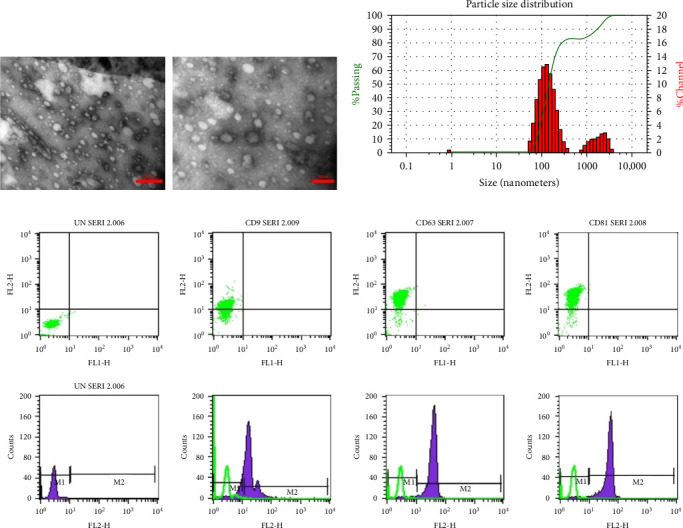
Characterization of MSC-Exo. (A) Morphology demonstrated by TEM. (B) DLS analysis showed that 82.9% of isolated vesicles were 128.5 nm on average. Most of the vesicles were in the range defined for exosomes (40–150 nm). (C) Flow cytometry histogram surface of exosomes for the CD63, CD81, and CD9 markers. Green histograms indicate exosomes and beads without specific markers, whereas blue histograms indicate the signals for each specific marker. Scale bar: 300 and 150 nm. DLS, dynamic light scattering; MSC-Exo, mesenchymal stem cell–derived exosomes; TEM, transmission electron microscopy.

The image of TEM showed that the exosomes were spherically shaped vesicles within the typical size range of 40–150 nm. Flow cytometry of surface markers CD63, CD81, and CD9 confirmed the exosomal nature of the isolated vesicles. The DLS test showed that 82.9% of isolated vesicles were 128.5 nm on average (Figure 3).

### 3.3. Gene Expression

**Figure 4:**
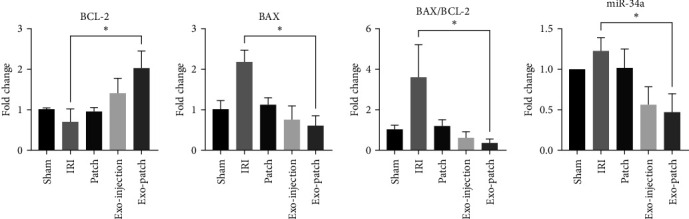
The expression of BCL-2, BAX, BAX/BCL-2, and miR-34a in animals treated with different experimental conditions. BAX, BCL-2-associated X (BAX); BCL-2, B-cell lymphoma-2; Exo, exosome; IRI, ischemia–reperfusion injury. Data are presented as the mean ± SD. *⁣*^*∗*^*p* < 0.05.

Statistical comparisons showed that the expression of apoptotic genes including miR-34a and its target BAX gene increased, while the expression of antiapoptotic target BCL-2 gene decreased in the IRI group (Figure 4). By applying different treatments, amelioration in the expression of the apoptotic gene and increment in the BCL-2 gene was observed. However, the changes in the genes only were significant in the Exo-patch group (*p* value BCL-2 = 0.0479, BAX = 0.0311, and miR-34a = 0.0224). The calculation of the BAX/BCL-2 ratio showed a significant decrement in the Exo-patch treatment group (*p* value = 0.0156).

### 3.4. The Changes in ROS and LPO Production

**Figure 5:**
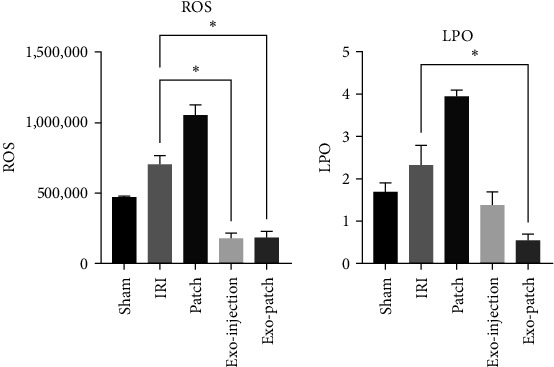
Kidney biomarkers of oxidative stress including ROS and LPO. Exo, exosome; IRI, ischemia–reperfusion injury; LPO, lipid peroxidation; ROS, reactive oxygen species. Data are presented as the mean ± SD. *⁣*^*∗*^*p* < 0.05.

The amount of ROS and LPO production was increased in the IRI group; however, in the patch group, the production of ROS and LPO notably increased (Figure 5). Treatment with Exo-injection (*p* value = 0.0219) and Exo-patch (*p* value = 0.0499) significantly ameliorated ROS production in IRI mice. Additionally, the amount of LPO decreased in the Exo-injection and Exo-patch groups. Decrement in the Exo-patch group was significant (*p* value = 0.0289).

### 3.5. Exo-Patch Ameliorates IRI Renal Dysfunction and Tissue Damage

**Figure 6:**
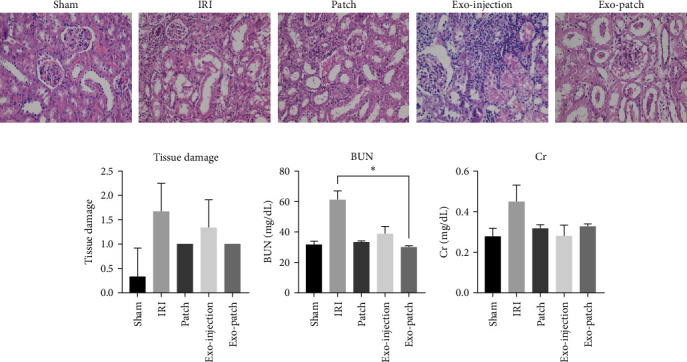
(A) Histological changes were examined by H&E staining (×400). (B) Tissue damage chart was based on histopathologic scores. (C, D) Measurement of BUN and Cr levels of the animals treated with the different experimental conditions. BUN, blood urea nitrogen; Cr, creatinine; Exo, exosome; H&E, hematoxylin and eosin; IRI, ischemia–reperfusion injury. Data are presented as the mean ± SD. *⁣*^*∗*^*p* < 0.05.

To confirm ischemic reperfusion-induced tissue injury, hematoxylin and eosin (H&E) staining was performed. Kidneys from the IRI group indicated extensive tubular injury that was determined with tubular dilation, vacuolization, cast formation, and loss of brush border. The treatment with Exo-patch and Exo-injection decreased the tissue damage; however, the decrement was not significant (Figure 6A,B). BUN and Cr levels were notably increased in the IRI group, and treatment with Exo-patch decreased BUN and Cr levels in IRI mice; however, decrement was only significant in BUN test (*p* value = 0.0211) (Figure 6C,D).

## 4. Discussion

Renal IRI remains a significant challenge in clinical medicine due to its propensity to induce cellular damage and apoptosis [19]. Novel therapeutic approaches are continuously sought to improve patient outcomes. Studies have demonstrated that MSC-Exo can improve tissue damage and enhance functional recovery after IRI [20]. However, direct injections of exosomes may result in a rapid dispersal of exosomes, potentially limiting their effectiveness.

Recent research applied microneedle patches to support the long-term release of MSC-Exo to improve tissue repair in medical conditions [21]. Indeed, one of the most prominent advantages of utilizing microneedle patches is the precision they offer in delivering therapeutic agents. The microneedles, typically smaller than a millimeter in length, enable precise targeting of the treatment area [18]. In the context of renal ischemia–reperfusion, this means the exosomes can be delivered directly to the affected renal tissue, enhancing their therapeutic efficacy. Traditional injection methods come with inherent risks, such as infection, tissue damage, or adverse reactions at the injection site. Microneedle patches, on the other hand, are associated with a lower risk of complications. They create micropores in the tissue, which generally heal quickly without significant adverse effects [22, 23].

Microneedles offer not only an efficient means of drug delivery but also a feature that sets them apart absorption into the tissue post-application [24]. This absorbability is a significant advantage, as it eliminates the need for needle removal and minimizes the risk of contamination and injury. Microneedles dissolve or degrade within the skin or tissue over time, leaving behind no trace of their presence. This attribute contributes to the overall patient experience, making microneedle-based therapies not only effective but also remarkably convenient and safe [24].

Microneedle patches offer a controlled release mechanism for therapeutic agents. This controlled release can be especially beneficial when delivering exosomes, as it ensures a steady and sustained presence of these bioactive molecules at the target site [21]. This can be especially important in managing chronic conditions like renal IRI where repeated treatments may be necessary. In contrast, traditional injections may result in a rapid dispersal of exosomes, potentially limiting their effectiveness.

Therefore, in this study, we investigated the potential therapeutic benefits of MSC-Exo delivered via the development of microneedle patches, focusing on their impact on the expression of key genes involved in apoptosis and cellular regulation.

Like other researches [10, 25], our findings indicated noteworthy alterations in the gene expression of BAX, BCL-2, and miR-34a following treatment with Exo-injection and Exo-patch. However, the significant downregulation of BAX, a proapoptotic gene, and upregulation of BCL-2, an antiapoptotic gene, in renal IRI tissue was only observed after Exo-patch treatment. In addition, the notable reduction of BAX/BCL-2 ratio as a key regulator of apoptosis suggests a potential prosurvival effect mediated by Exo-injection and Exo-patch. This aligns with previous studies [11, 14] indicating the protective effects of exosomes in various models of tissue injury. The decrement of miR-34a expression was particularly intriguing in the Exo-patch group, as miR-34a is known to play a role in promoting apoptosis and cell cycle arrest. Like previous study [26], the downregulation of miR-34a may indicate a regulatory mechanism by which exosomes mitigate cellular stress and apoptosis in renal tissue following ischemia–reperfusion. Furthermore, the downregulation of miR-34a may represent a key mechanistic insight into the cytoprotective actions of exosomes. miR-34a is known to target and inhibit the expression of genes involved in cell survival and DNA repair [27]. Therefore, the suppression of miR-34a by exosomes may mitigate the detrimental effects of renal IRI by preserving cellular integrity and function.

Our study revealed that the utilization of microneedle patches for the targeted delivery of MSC-Exo had a profound impact on oxidative stress markers. ROS and LPO levels were significantly reduced in the renal tissue treated with Exo-patch. These findings underline the potential antioxidant properties of exosomes and their capacity to mitigate the damaging effects of oxidative stress in renal cells, which is a pivotal factor in the pathophysiology of renal IRI [28]. Following the insertion of microneedles on the renal cortex of the kidneys, neutrophile recruitment occurs. Neutrophils are the key effectors of the inflammatory cascade resulting in the generation of ROS, chemotaxis, and phagocytosis. Therefore, it seemed that by the insertion of free microneedles, ROS and LPO levels were increased, while in the Exo-patch group, the presence of MSC-derived exosomes resulted in a significant reduction in ROS and LPO levels.

Evaluation of renal function through the BUN and Cr is crucial in assessing kidney injury and function. Our study demonstrated that treatment with Exo-patch led to a decrement in the BUN and Cr levels compared to the IRI group, suggesting that exosome therapy may have a renoprotective effect by ameliorating renal dysfunction following IRI [10, 29]. Inflammation and the formation of tubular casts are prominent features of renal IRI. In our study, a marked reduction in inflammatory markers and a decrease in tubular cast formation were observed in the renal tissue treated with Exo-patch. This finding indicates that exosome therapy not only exerts an anti-inflammatory effect but also mitigates the structural damage caused by tubular cast formation. The suppression of inflammation and tubular cast damage is crucial in preserving renal function and preventing long-term kidney damage, like Long Li et al. [10] said.

These additional observations further emphasize the multifaceted benefits of using microneedle patches for the delivery of exosomes in the context of renal IRI. On the other hand, free microneedle patches were composed of HA, and as it was reported, HA is a biocompatible and biodegradable natural polysaccharide which is the principal ligand of CD44 and is significantly important in the development of nanoparticles with a preferential for CD44-overexpressed tissue accumulation and increased cellular uptake [30]. Therefore, selecting HA as a targeting biomaterial for IRI is meaningful and practical, considering the overexpression of CD44 receptors in AKI models. In addition, it was reported that HA-based nanoparticles could relieve renal tubular apoptosis and exert an antiapoptotic effect, contributing to renal recovery [30]. Although the beneficial effects of HA in various scaffolds have been mentioned, further investigation was needed in the structure of the microneedle patch produced by our group. Therefore, for more certainty and a more detailed investigation, we studied the patch group alone as a group to evaluate its effects alone. The result demonstrated free microneedles themselves can have a positive effect on IRI, while this effect becomes significantly and synergistically highlighted when exosomes are added. Moreover, the final microneedles had PVA as the backing layer because of taking its advantage to form more uniform microneedles with optimal flexibility and better application during the insertion in the kidney [31]. Previous studies mentioned that PVA-based hydrogels are widely used in tissue engineering, owing to their high-water content and porous microstructures, which can facilitate cell growth and tissue regeneration [31]. Our result showed that free microneedle patches improved tissue damage and reduced BUN and Cr compared to the IRI group which means that free microneedles containing PVA as the backing layer did not show any significant side effects for kidney tissue. Besides, the free microneedle patch caused the increase in antiapoptotic BCL-2 gene expression and decreased the BAX and miR-34a expression which play an important role in promoting apoptosis and cell cycle arrest. Thus, our findings support the idea that PVA did not show any significant side effects for kidney tissue.

**Figure 7:**
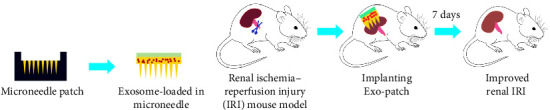
The effect of exosome loaded in microneedle patch on renal ischemia–reperfusion injury in a mouse model. (A) Exosome microneedle patch preparation. (B) Induction of renal ischemic reperfusion injury in a mouse model and treatment with Exo-patch. Exo, exosome; IRI, ischemic reperfusion injury.

Overall, by addressing oxidative stress, improving renal function, and reducing inflammation and structural damage, applying a microneedle patch for the delivery of exosomes showed great promise as a comprehensive and effective strategy for renal protection and recovery (Figure 7). However, it is suggested to study different doses of exosome loaded in microneedle patch on different days for more detailed investigation. The clinical implications of our findings are promising. Administration of MSC-Exo via microneedle patch could serve as a minimally invasive, controlled release and open the door to a novel and patient-centered approach for managing renal IRI [15]. However, further research is warranted to validate these results in larger animal models and, ultimately, in clinical trials.

## 5. Conclusion

In conclusion, our study highlights the potential advantages of utilizing microneedle patches for the delivery of MSC-Exo in the context of renal IRI. This innovative approach offers precise, noninvasive, and patient-friendly delivery, with the potential to improve therapeutic outcomes and patient experiences. As we continue to explore and refine this method, we anticipate significant advancements in the treatment of renal IRI and other medical conditions.

## Data Availability

The data that support the findings of this study are available from the corresponding author upon reasonable request.
